# Supplementary material for: Women’s Intentions to Engage in Risk-Reducing Behaviours after Receiving Personal Ovarian Cancer Risk Information: An Experimental Survey Study
Source: Cancers (Basel). 2020 Nov 27;12(12):3543. doi: 10.3390/cancers12123543 (PMC7760356; doi:10.3390/cancers12123543)
Supplement: Supplementary file 1 [file cancers-12-03543-s001.pdf]

Supplementary Materials: Women's Intentions to Engage in Risk-Reducing Behaviours after Receiving Personal Ovarian Cancer Risk Information: An Experimental Survey Study

Ailish Gallagher, Jo Waller, Ranjit Manchanda, Ian Jacobs and Saskia Sanderson

Table S1. Data for Figure 3.

Behavioural Intention	*n* (%)			
	Hypothetical Result Received			
	5% SNPs & Lifestyle Factors	10% SNPs and Lifestyle Factors	10% BRCA2 Mutation	Total
Risk-reducing surgery	95 (27.9)	90 (26.2)	105 (31.4)	290 (28.5)
Surveillance	111 (32.6)	112 (32.7)	122 (36.5)	345 (33.9)
Lifestyle	71 (20.9)	86 (25.1)	56 (16.8)	213 (20.9)
Do Nothing	12 (3.5)	5 (1.5)	6 (1.8)	23 (2.3)
Not Sure	51 (15.0)	50 (14.6)	45 (13.5)	146 (14.4)

Table S2. Mean EPPM variables by experimental group.

EPPM Variables	Mean (SD)		
	Group 1<br>5% SNPs & Lifestyle Factors	Group 2<br>10% SNPs and Lifestyle Factors	Group 3<br>10% Rare Genetic Variant
Perceived Severity	4.54 (0.58)	4.51 (0.60)	4.51 (0.57)
Perceived Response Efficacy of RRSO	4.03 (0.84)	4.01 (0.76)	4.06 (0.75)
Perceived Response Efficacy of TVU	3.64 (0.89)	3.61 (0.86)	3.68 (0.81)
Perceived Risk	3.51 (0.79)	3.46 (0.86)	3.57 (0.80)
Self-Efficacy for RRSO surgery	3.05 (1.38)	2.91 (1.40)	2.96 (1.24)
Perceived Control	2.54 (0.85)	2.47 (0.84)	2.49 (0.89)

EPPM: Extended Parallel Processing Model.

© 2020 by the authors. Licensee MDPI, Basel, Switzerland. This article is an open access article distributed under the terms and conditions of the Creative Commons Attribution (CC BY) license (http://creativecommons.org/licenses/by/4.0/).
